# The role of personality traits in mediating the relation between fear of negative evaluation and social interaction anxiety

**DOI:** 10.3389/fpsyg.2023.1268052

**Published:** 2023-10-19

**Authors:** Crenguța Mihaela Macovei, Ștefania Bumbuc, Fabiana Martinescu-Bădălan

**Affiliations:** Department of Applied Social Sciences and Humanities, “Nicolae Bălcescu” Land Forces Academy, Sibiu, Romania

**Keywords:** fear of negative evaluation, social interaction anxiety, personality traits, personality facets, serial mediation

## Abstract

**Introduction:**

Social interaction anxiety and fear of negative evaluation have many maladaptive outcomes and, in order to counteract their effects, it is essential to identify those psychological or social factors that make people vulnerable to them. One of these factors is the individual’s personality structure: some personality traits increase the individuals’ vulnerability to symptoms of social anxiety, while others protect them.

**Methods:**

The aim of this paper is to analyse the role of HEXACO personality traits in mediating the relationship between fear of negative evaluation and social anxiety, in a sample of 352 cadets from the Land Forces Academy of Sibiu. The relationships between these concepts were analysed using structural equation modeling (SEM) in several hypothetical models, two of which were ultimately validated.

**Results:**

In the first model, the fear of negative evaluation has an indirect effect on social interaction anxiety through the mediation of extraversion, conscientiousness, and altruism, separately. Furthermore, extraversion, conscientiousness, and altruism play a serial mediating role in the association between the fear of negative evaluation and social interaction anxiety. In the second model, the fear of negative evaluation has an indirect effect on social interaction anxiety through the mediation of social boldness, liveliness, and organization, separately, but not through altruism. Social boldness, liveliness, and organization played a serial mediating role in the relationship between the two constructs, while altruism moderated the relationship between liveliness, organization, and social interaction anxiety.

**Discussion:**

Analysing the relationship between the individuals’ personality traits, social anxiety, and fear of negative evaluation facilitated the identification of ways to cultivate desirable behaviours in social environments typified by compliance, discipline, uniformity, and rigor.

## 1. Introduction

Social anxiety is one of the most commonly experienced forms of anxiety; throughout their life, most individuals have experienced low to moderate anxiety symptoms in various social situations. Their role is to energize the individual in social interactions or situations where he/she has to perform in front of or with others, but they must dissipate after the interactions or situations have ceased. If they do not disappear, these symptoms end up profoundly affecting ever larger areas of the individual’s life.

Certain cognitive models support the idea that social anxiety is generated to a significant extent by fears of perceived negative evaluation; as a latent construct, fear of negative evaluation is partially inherited and is closely correlated with trait anxiety, social avoidance, general fears and psychopathologies (Carleton et al., 2007).

Therefore, fear of negative evaluation and social interaction anxiety are two strongly correlated constructs and they have many maladaptive outcomes: depression (Wang et al., 2012; Kim and Duval, 2022), poor academic adjustment (Archbell and Coplan, 2022) alcohol-related problems and alexithymia (Lyvers et al., 2018; Kumar et al., 2022; Caumiant et al., 2023), interpersonal relationships problems (Tonge et al., 2020; Alexandru, 2022) isolation and social avoidance (Lee et al., 2022; Lyngdoh et al., 2022; Chen et al., 2023). Recently, increasingly extensive use of social media, connectivity, and high digital visibility are associated with high levels of social anxiety (Lee-Won et al., 2015; Carruthers et al., 2019; Rad et al., 2019, 2020). Fayaz et al. (2021), for example, show that people with an intense fear of negative evaluation become compulsive social media users.

Personality traits play significant roles in the emergence, maintenance, and development of social anxiety throughout an individual’s life (Costache et al., 2020; Chung et al., 2022). Some personality traits facilitate the onset of social anxiety symptoms, while others protect the individual from them. Studies that analyze the relationship between personality traits, social interaction anxiety and fear of negative evaluation are relatively few, because most of the researchers prefer to focus on analyzing the role of traits in the etiology of anxiety disorders. Therefore, our study set out as its main objective to examine this relationship; since social interaction anxiety and fear of negative evaluation are strongly correlated, we set out to analyze the possibility that personality traits play a mediating role between the two, using structural equation modeling (SEM) as the method of statistical analysis.

This research used the HEXACO model of personality developed by Ashton and Lee (2007), which consists of six personality dimensions, namely honesty-humility (H), emotionality (E), extraversion (X), agreeableness (A), conscientiousness (C) and openness to experience (O), to which the interstitial trait called altruism (versus antagonism) has been added.

In order to determine which of the six personality traits could be entered into the structural model, a hierarchical regression analysis was performed, with social interaction anxiety as a criterion variable and the fear of negative evaluation, the six personality traits and the interstitial facet of altruism as independent variables. The results indicated the fact that only extraversion, conscientiousness and altruism predict the level of social interaction anxiety, after controlling the influence of the fear of negative evaluation. These were the variables entered in the SEM analysis.

As personality traits, extraversion and conscientiousness each consist of four facets. The extraversion facets are: social self-esteem, social boldness, sociability, and liveliness. The facets associated with conscientiousness are: organization, diligence, perfectionism, and prudence. In the next stage of the research, we set out to analyze the possibility that these facets also play a mediating role in the relationship between social interaction anxiety and fear of negative evaluation. In order to determine which of these facets could be included in an appropriate structural model, a hierarchical regression analysis was performed, with social interaction anxiety as criterion variable and the fear of negative evaluation, the eight facets of personality listed above and the interstitial facet of altruism as independent variables. The results indicated the fact that only social boldness, liveliness, organization, and altruism predict the level of social interaction anxiety, after controlling the influence of the fear of negative evaluation. These were the variables entered in the SEM analysis.

During data processing, several possible models of the relationship between these variables were proposed and tested. The two models that met the validity criteria are presented in this paper.

## 2. Social interaction anxiety

Social anxiety can manifest in two forms: as social interaction anxiety, when the individual experiences anxiety when interacting with others in different social situations (eating, walking, talking etc), or as performance anxiety, when they are being observed and evaluated by others while performing a task (Mattick and Clarke, 1998). A person may feel anxious in one or both types of situations. Individuals with a very high level of social interaction anxiety usually receive the diagnosis of social anxiety disorder, which implies an intense fear of social interactions or of situations involving observation by others (Heimberg et al., 1992). One model that explains very clearly the psychological processes underlying social anxiety disorder is the cognitive model of social anxiety proposed by Clark and Wells (1995). The model describes six factors that perpetuate social anxiety:

1. *Maladaptive social-evaluative beliefs*; these are of three types: (a) high standards for social performance (b) conditional beliefs regarding social evaluation and (c) unconditional beliefs about the self;
2. *Self-focus attention*: the anxious person turns their attention inward to monitor their physical reactions, emotions, and thoughts in social situations or when they have to solve a task in front of others; with people suffering from social anxiety, this mechanism results in the accentuation of negative beliefs about their social performance;
3. *Attention toward threat in the environment* refers to the tendency of anxious people to detect threatening stimuli very quickly (facilitation bias), to remain anchored to them (difficulty of disengagement) or to move away quickly after detecting them (avoidance bias);
4. *Anticipatory processing* occurs when the anxious person experiences intrusive and persistent negative thoughts about themselves and their past failures, expects rejection from others before a social event or poor performance before solving a task in front of others;
5. *Post-event processing* is a form of rumination that refers to the retrospective analysis of one’s own performance in social situations; in the case of anxious people, this analysis is vitiated by maladaptive beliefs about oneself and one’s own performance (Katz et al., 2019);
6. *Safety behaviors* are mainly mental strategies by which the anxious person tries to prevent the consequences they fear from occurring or to minimize their effects. They are not only ineffective, but also harmful in the long term, because “they prevent the individual from discovering that the feared outcome was unlikely and/or not catastrophic; they intensify self-focus; they may increase feared symptoms; they can draw attention to feared symptoms; and they can also interfere with the social interaction” (Leigh et al., 2021, p. 2).

The six factors described above can affect an individual’s social and interpersonal behavior in different proportions and combinations, even if the individual does not have a diagnosis of social anxiety disorder (Sagliano et al., 2014; Heeren et al., 2020; Seinsche et al., 2023).

## 3. Fear of negative evaluation

Fear of negative evaluation is included in all studies as a main indicator of social anxiety (Weeks et al., 2010; Van der Molen et al., 2014; Preston et al., 2023). It is a psychological state characterized by an intense fear of being judged, criticized, humiliated or just perceived negatively by others, “apprehension about others’ evaluations, distress over their negative evaluations, avoidance of evaluative situations and the expectation that others would evaluate oneself negatively” (Watson and Friend, 1969, p. 449).

Individuals with high levels of fear of negative evaluation often experience extreme anxiety and discomfort in social situations, which triggers avoidance behaviors or intense stress (Reichenberger et al., 2018). As symptoms, individuals may experience a range of unpleasant states physically (Weeks and Zoccola, 2016), intellectually (Maresh et al., 2017), emotionally, and behaviorally (Takagishi et al., 2016). Physical symptoms can include rapid heartbeat, sweating, trembling, nausea, and difficulty breathing. Emotionally, people may feel intense fear, embarrassment, or self-consciousness. Behaviorally, they may avoid social situations, struggle with public speaking, or have difficulty initiating or maintaining conversations.

The intensity and modes of manifestation of the fear of negative evaluation may be influenced by educational factors (Downing et al., 2020; Salazar-Ayala et al., 2021; Busch et al., 2023), by the individual’s life experiences (Liu T. et al., 2022; Lucero et al., 2022), and by cultural and societal norms that place a high value on social acceptance and conformity (Vaswani et al., 2022).

In the context of professional life, fear of negative evaluation is positively correlated with interview anxiety and social-evaluative workplace anxiety (Zhang et al., 2022). In educational contexts, some research results indicate significant differences between women and men, as well as between undergraduate and graduate students. Thus, “female students showed more fear of negative evaluation and social anxiety than male students; similarly, undergraduate students showed more social anxiety” (Iqbal and Ajmal, 2018, p. 49).

## 4. Personality traits and social anxiety

Social anxiety has a positive relationship with neuroticism and is negatively associated with extraversion while its relationship with conscientiousness, agreeableness, and openness to experience is a mixed one.

Neuroticism is a strongly genetically determined personality trait that predisposes the individual to the development of mood disorders or social anxiety disorder or to the manifestation of symptoms specific to them but at a non-clinical level (Scaini et al., 2014; Stein et al., 2017; Vinograd et al., 2020). Newby et al. (2017) found that self-consciousness, vulnerability, and impulsiveness as facets of neuroticism predict interaction anxiety. Allan et al. (2017) studied the role of vulnerabilities to emotional distress in the relationship between neuroticism and social anxiety and found that inhibitory intolerance of uncertainty, fear of negative evaluation, and anxiety sensitivity to social concerns significantly influence this relationship. Also, in Clague and Wong's (2023) study, neuroticism was positively associated with social-evaluative beliefs, self-focus, and post-event processing as components of social anxiety.

Extraversion is generally associated with low levels of social anxiety (Watson et al., 2005; Naragon-Gainey et al., 2009). Duffy et al. (2018) show that people with a high level of extraversion expect and relate positively to changes in affective experience following social interaction, while people with a low level of extraversion have negative expectations from social interactions and anticipate negative evaluations from others and therefore avoid them. However, in the experiment conducted by these authors, participants “with the most fear about being negatively evaluated do better: their partners find them more skilled than their low-extraversion counterparts who do not share this fear of negative evaluation” (Duffy et al., 2018, p. 17). Therefore, people with a high level of fear of negative evaluation seem to put more effort into their social interactions in order not to give others reasons to evaluate them negatively.

Spinhoven et al. (2014) analyzed the relationship between lower level facets of extraversion (positive affectivity, sociability and activity), depression and social anxiety and found a significant association between low sociability, lack of positive affectivity and trait social anxiety. People with high levels of extraversion and openness have an increased tendency “to seek external support, actively reconstruct stressful events, provide alternative cognitive strategies for rumination, and form harmonious interpersonal relationships, and all these will make them less prone to anxiety” (Liu et al., 2023a, p. 2).

In the study conducted by Costache et al. (2020) neuroticism and extraversion had the highest ability to discriminate between the social anxiety disorder group and the control group of healthy individuals. These authors identified a low level of conscientiousness in patients with social anxiety disorder, a moderate correlation of agreeableness and conscientiousness with the psychopathology included in the study and the total lack of association between openness and anxiety and depression symptoms.

The combination of high levels of neuroticism and low levels of extraversion is found in many emotional disorders, including social anxiety disorder and depression (Naragon-Gainey et al., 2014). In the study conducted by Kaplan et al. (2015), social anxiety was positively correlated with neuroticism and negatively with extraversion, with confidence as a facet of agreeableness and with self-efficacy as a facet of conscientiousness; also, a high level of openness to experience was correlated with a decrease in the level of social anxiety associated with low trust.

In their meta-analytic study, Kotov et al. (2010) studied the relationship between the Big Five personality traits and specific depressive, anxiety, and substance use disorders (SUD) in adult samples and found that all groups with these disorders were characterized by high levels of neuroticism and low levels of conscientiousness. Many of these disorders involved low levels of extraversion while agreeableness and openness were very weakly or not at all associated with them.

Watson and Naragon-Gainey (2014) found that conscientiousness, agreeableness, and openness have a weak association with emotional disorders and have a very low contribution to predicting their symptoms, even when the influence of neuroticism and extraversion is controlled.

Regarding altruism, there are few studies relating this concept to social anxiety or fear of negative evaluation. Among the effects of altruism on individuals, Post (2005) mentions positive social integration, distraction from personal problems and from anxiety. It was demonstrated that individuals who display helping behavior (as a type of altruism) have a lower score of fear of negative evaluation than individuals who do not help in some situations (Karakashian et al., 2006).

## 5. Materials and methods

### 5.1. Participants and procedure

The sample consisted of 352 first-year students at “Nicolae Bălcescu” Land Forces Academy of Sibiu, 149 girls (42.32%) and 203 boys (57.67%), with ages between 18 and 21 years. All students enrolled in the discipline *Military Psycho-Sociology* in the first semester completed the scales used in this study as a part of a seminar activity between October and December 2022. Students were asked to give written consent for their responses to these scales to be processed and the results to be used in this article, with the assurance that responses would be anonymized. In order to better understand the processing of their answers, the students were briefly presented the statistical procedures used. Responses from students who did not give their consent were excluded from the processing.

### 5.2. Instruments

1. *Social Interaction Anxiety Scale (SIAS)* is a 20 items scale developed by Peters (2000) to measure social anxiety triggered by communication or interaction with other people that may involve scrutiny. The participants need to give their answers on a five-point Likert scale, from (0) Not at all characteristic or true of me to (4) Extremely characteristic or true of me. The Cronbach’s alpha coefficient for the entire scale was 0.92.
2. *Brief Fear of Negative Evaluation Scale* is a 12-items version of the original scale developed by D. Watson and R. Friend in 1969. This brief version was developed by Leary (1983) and uses a five-point Likert scale, from (1) Not at all characteristic of me to (5) Extremely characteristic of me. The Cronbach’s alpha coefficient for the entire scale was 0.84.
3. *Personality traits and facets* were measured using *HEXACO-100* personality inventory (Lee and Ashton, 2018), a 100-item measure using a five-point Likert scale, from (1) strongly disagree to (5) strongly agree. This inventory consists of six scales: honesty-humility (H), emotionality (E), extraversion (X), agreeableness (A), conscientiousness (C), and openness to experience (O) and an interstitial scale, entitled altruism (versus antagonism). The Cronbach’s alpha coefficient for the entire inventory was 0.88, and it ranged from 0.77 to 0.83 for the main six scales.

### 5.3. Data analysis approach

The statistical software package SPSS 28.0 with Amos 28.0 was used for data analysis. The relationships between these constructs were analyzed by structural equation modeling (SEM; ML method, 5,000 Bootstrap samples, 95% CI).

In order to determine the adequacy of the models, goodness-of-fit indexes and path coefficients were analyzed. In the first stage of structural equation modeling, a set of analyses was applied to determine the discriminant and convergent validity of the constructs included in the hypothesized models, according to the recommendations of Anderson and Gerbing (1988). A first analysis was aimed at identifying those items with an acceptable level of loading for social interaction anxiety, fear of negative evaluation and altruism scales. For the HEXACO personality traits scales, the Cronbach alpha and composite reliability coefficients were calculated.

The second analysis aimed at identifying those personality traits that have a mediating effect on the relationship between fear of negative evaluation and social interaction anxiety (first structural model), as well as those facets of the previously identified traits that also exert this mediating effect (second structural model). Having identified some defensible measurement models, SEM analyses were conducted to test the proposed relationships and assess them against fit indices proposed by Byrne (2006), Hu and Bentler (1999), and Schumacker and Lomax (2016).

## 6. Results

Table 1 presents the items retained in the scales measuring social interaction anxiety, fear of negative evaluation and altruism, together with alpha reliability (α), composite reliability (CR) and average variance extracted (AVE) for each scale. Items with loadings lower than 0.60 were removed from the scale so that the AVE value reached the acceptable threshold of 0.50 (Peterson et al., 2020).

**Table 1:** Items loadings, composite reliability (CR), average variance extracted (AVE) and alpha Cronbach (α) for the scales measuring social interaction anxiety, fear of negative evaluation and altruism.

Scales	Item loading	CR	AVE	*α*
Social Interaction Anxiety (SIA)		0.94	0.50	0.93
1. I have difficulty making eye-contact with others.	0.750			
2. I find it difficult mixing comfortably with the people I work with.	0.694			
3. I tense- up if I meet an acquaintance in the street.	0.782			
4. When mixing socially, I am uncomfortable	0.735			
5. I feel tense if I am alone with just one person.	0.728			
6. I have difficulty talking with other people.	0.720			
7. I worry about expressing myself in case I appear awkward.	0.794			
8. I find it difficult to disagree with another’s point of view.	0.657			
9. I have difficulty talking to a potential romantic partner.	0.709			
10. I find myself worrying that I will not know what to say in social situations.	0.797			
11. I am nervous mixing with people I do not know well.	0.698			
12. I feel I’ll say something embarrassing when talking.	0.811			
13. When mixing in a group, I find myself worrying I will be ignored.	0.741			
14. I am tense mixing in a group.	0.828			
Fear of negative evaluation (FNE)		0.92	0.62	0.92
1. I worry about what other people will think of me even when I know it does not make any difference.	0.778			
2. I am frequently afraid of other people noticing my shortcomings.	0.794			
3. I am afraid others will not approve of me.	0.749			
4. I am afraid that people will find fault with me.	0.786			
5. When I am talking to someone, I worry about what they may be thinking about me.	0.884			
6. I am usually worried about what kind of impression I make.	0.695			
7. I often worry that I will say or do the wrong things.	0.852			
Altruism (Alt)		0.80	0.57	0.62
1. I try to give generously to those in need.	0.681			
2. It would not bother me to harm someone I did not like. (R)	0.747			
3. People see me as a hard-hearted person. (R)	0.841			

Items indicated with R are reverse-keyed items.

### 6.1. First structural model

In order to determine which of the personality traits could be entered into the structural model, a hierarchical regression analysis was performed, with social interaction anxiety as a criterion variable and the fear of negative evaluation, the six personality traits and the interstitial facet of altruism as independent variables.

In the first step, *fear of negative evaluation* accounted for 52% of the variance and the model was significant [*F* (1,350) = 387.64, *p* < 0.001], with *fear of negative evaluation* as significant predictor (*β* = 0.72, *p* < 0.001).

By adding the *personality traits* as independent variable in the second step of the regression model, and by controlling the influence of the *fear of negative evaluation*, the predictive value of the second model increased to 61% (ΔR^2^ = 0.084; *F* (2,348) =37.47, *p* < 0.001), with *fear of negative evaluation* (*β* = 0.58, *p* < 0.001), *extraversion* (*β* = −0.22, *p* < 0.001) and *conscientiousness* (*β* = −0.14, *p* < 0.001) as significant predictors.

In the third step, *altruism* was added to the regression model resulting in an increase of the predictive value of the model to 62% (ΔR^2^ = 0.016; *F* (1,347) = 15.19, *p* < 0.001), after controlling the influence of *the fear of negative evaluation* and *personality traits*, with *altruism* (*β* = −0.13, *p* < 0.001), *fear of negative evaluation* (*β* = 0.57, *p* < 0.001), *extraversion* (*β* = −0.21, *p* < 0.001) and *conscientiousness* (*β* = −0.10, *p* = 0.008) as significant predictors. As observed, the power of conscientiousness to predict the level of social interaction anxiety decreases when altruism is introduced in the model as an independent variable. Therefore, the above-mentioned psychological constructs have been introduced into the SEM model. Considering the values provided by the hierarchical regression analysis, the following hypotheses regarding the relationships between the measured constructs were formulated:

*H1*: Fear of negative evaluation is positively associated with social interaction anxiety.

*H2*: Fear of negative evaluation is negatively associated with extraversion, conscientiousness, and altruism.

*H3*: Extraversion is positively associated with conscientiousness.

*H4*: Conscientiousness is positively associated with altruism.

*H5*: Extraversion, conscientiousness, and altruism are negatively associated with social interaction anxiety.

*H6*: Extraversion, conscientiousness, and altruism play a serial mediating role in the association between fear of negative evaluation and social interaction anxiety.

Table 2 shows the means, standard deviation, and correlations between the variables included in this model.

**Table 2:** Correlation matrix between social interaction anxiety, fear of negative evaluation, extraversion, conscientiousness, and altruism.

	Variables	*M*	SD	1	2	3	4	5
1	Social interaction anxiety (SIA)	0.87	0.82	(0.93)				
2	Fear of negative evaluation (FNE)	0.60	0.67	0.725^**^	(0.92)			
3	Extraversion (Ext)	3.80	0.50	−0.536^**^	−0.409^**^	(0.82)		
4	Conscientiousness (Con)	3.91	0.47	−0.444^**^	−0.322^**^	0.496^**^	(0.81)	
5	Altruism (Alt)	4.16	0.68	−0.370^**^	−0.239^**^	0.271^**^	0.354^**^	(0.62)

*N* = 352; Extraversion, Conscientiousness = HEXACO Personality Traits; Altruism = HEXACO Interstitial personality facet; Values of the internal consistency alphas are displayed in round brackets. *p < 0.05. **p < 0.01. The composite reliability coefficient of both extraversion and conscientiousness scales is 0.87.

In the hypothesized model, fear of negative evaluation was entered as the independent variable (IV), social interaction anxiety was entered as the dependent variable (DV), extraversion was entered as first mediator (M1), conscientiousness was entered as second mediator (M2), and altruism was entered as third mediator (M3). According to this model, there are five possible pathways linking fear of negative evaluation to social interaction anxiety – one direct path and four indirect paths. As expected, the direct path linked fear of negative evaluation to social interaction anxiety. The first indirect pathway was through extraversion (M1), the second indirect pathway was through conscientiousness (M2) and the third indirect pathway was through altruism (M3). The fourth indirect pathway was through extraversion (M1), conscientiousness (M2) and altruism (M3), in serial.

**Figure 1:**
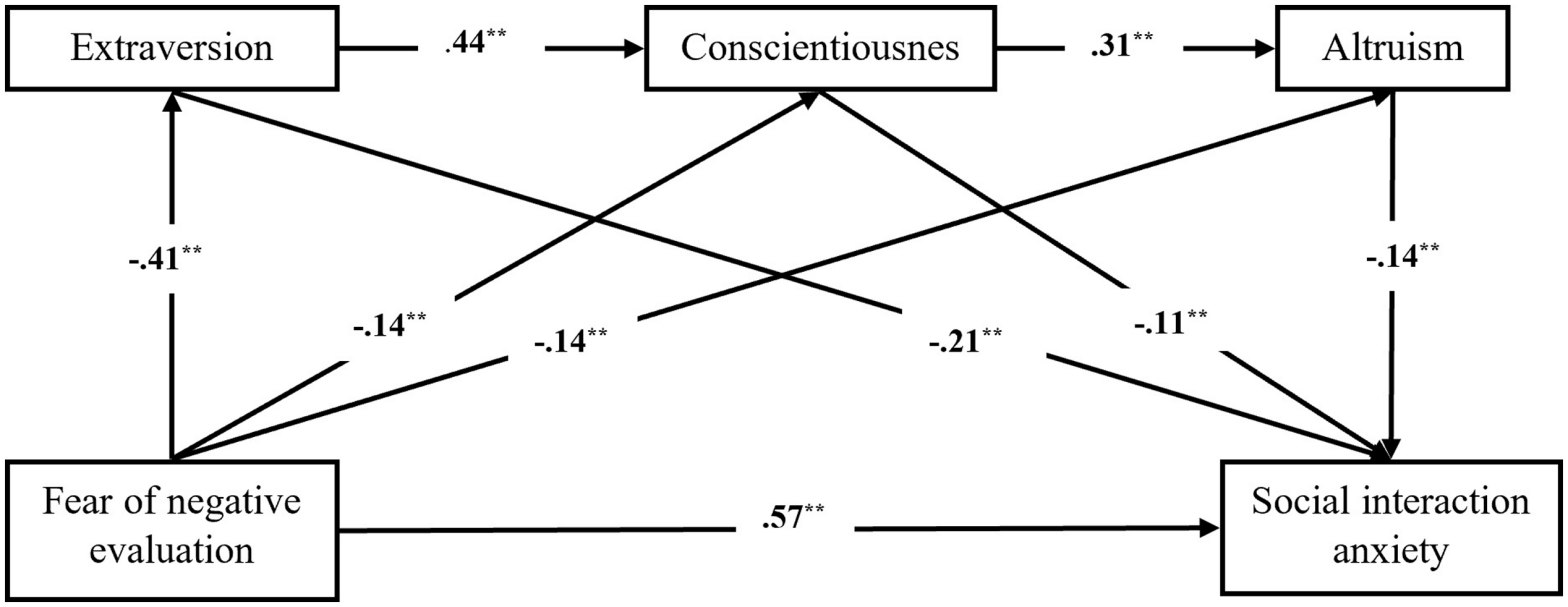
The path diagram of the relationships between fear of negative evaluation, extraversion, conscientiousness, altruism, and social interaction anxiety.

All the values of the tested model reached the suggested values, indicating that the model is adequate (Table 3). The validated model is presented in Figure 1.

**Table 3:** Goodness-of-fit of the first structural model.

Fit index	*X*^2^/df	IFI	CFI	TLI	GFI	NFI	RFI	AGFI	SRMR	RMSEA
Suggested value	0–3	> 0.90	> 0.95	> 0.95	> 0.95	> 0.95	> 0.90	> 0.90	< 0.08	< 0.06
Values of this study	2.18	0.99	0.99	0.97	0.99	0.99	0.96	0.96	0.016	0.058

### 6.2. Hypotheses tested

As illustrated in Table 4, the hypotheses H1 to H5 and the relationships paths were supported by the SEM results. Therefore:

- Fear of negative evaluation significantly and positively predicted social interaction anxiety (*β* = 0.57, *p* < 0.001), hence H1 was verified;
- Fear of negative evaluation significantly and negatively predicted extraversion (*β* = −0.41, *p* < 0.001), conscientiousness (*β* = −0.14, *p* = 0.004), and altruism (*β* = −0.14, *p* = 0.008), therefore H2 was supported;
- Extraversion was positively associated with conscientiousness (*β* = 0.44, *p* < 0.001) and conscientiousness was positively associated with altruism (*β* = 0.31, *p* < 0.001), therefore H3 and H4 were supported;
- Extraversion (*β* = −0.21, *p* < 0.001), conscientiousness (*β* = −0.11, *p* = 0.007), and altruism (*β* = −0.14, *p* < 0.001) significantly and negatively predicted social interaction anxiety, hence H5 was supported.

**Table 4:** The test results of path relationships between fear of negative evaluation, social interaction anxiety, extraversion, conscientiousness, and altruism.

Hypothesis	Path	Unstandard estimates	S.E.	C.R.	Sig.	Standard estimates	LL 95%CI	UL95%CI	Hypothesis test
H1	FNE--->SIA	0.466	0.030	15.634	***	0.572	0.493	0.640	Supported
H2	FNE--->Ext	−0.251	0.030	−8.389	***	−0.409	−0.494	−0.315	Supported
	FNE--->Con	−0.083	0.029	−2.859	0.004	−0.144	−0.243	−0.035	
	FNE--->Alt	−0.117	0.044	−2.672	0.008	−0.139	−0.255	−0.026	
H3	Ext--->Con	0.412	0.047	8.713	***	0.437	0.345	0.524	Supported
H4	Con--->Alt	0.446	0.075	5.923	***	0.309	0.195	0.410	Supported
H5	Ext--->SIA	−0.281	0.052	−5.380	***	−0.213	−0.304	−0.128	Supported
	Con--->SIA	−0.149	0.056	−2.682	0.007	−0.106	−0.177	−0.029	
	Alt--->SIA	−0.135	0.034	−3.932	***	−0.139	−0.221	−0.053	

FNE, Fear of negative evaluation; SIA, Social interaction anxiety; Ext, Extraversion; Con, Conscientiousness; Alt, Altruism.

Based on these results, we can conclude that fear of negative evaluation can have an indirect effect on social interaction anxiety through the mediation of extraversion, conscientiousness, and altruism, separately.

To test H6, which states that extraversion, conscientiousness, and altruism play a serial mediating role in the association between fear of negative evaluation and social interaction anxiety, the mediation chain FNE--->Ext--->Con--->Alt--->SIA has been specified and tested in Amos 28.0, using the Bootstraping procedure (95%CI, 5,000 random samples). The results were as follows: *β = 0*.007, BootLL = 0.002, BootUL = 0.013, *p* < 0.001. Based on these results, it can be stated that extraversion, conscientiousness, and altruism play a serial mediating role in the association between fear of negative evaluation and social interaction anxiety; therefore, H6 was supported. The values of the effects of indirect path relationships of this model are presented in Table 5.

**Table 5:** Bootstrap analysis of the significance test of the intermediate model effects in the first model.

	Path	Standard indirect effect	Unstandardized indirect effect	*p*	Bootstrap (95% CI)	
					Lower Limit	Upper Limit
Intermediary effect	FNE--->Ext--->SIA	0.086	0.071	<0.001	0.043	0.107
	FNE--->Con--->SIA	0.015	0.012	0.008	0.002	0.031
	FNE--->Alt--->SIA	0.019	0.016	0.010	0.002	0.040
	FNE--->Ext--->Con--->Alt--->SIA	0.007	0.006	0.001	0.002	0.013
Total intermediation effect		0.127	0.105		0.049	0.191

FNE, Fear of negative evaluation; SIA, Social interaction anxiety; Ext, Extraversion; Con, Conscientiousness; Alt, Altruism.

### 6.3. Second structural model

In order to determine which facets of extraversion (social self-esteem, social boldness, sociability, and liveliness) and conscientiousness (organization, diligence, perfectionism, and prudence) could be included in an appropriate structural model, a hierarchical regression analysis was performed, with social interaction anxiety as criterion variable and the fear of negative evaluation, the eight facets of personality listed above and the interstitial facet of altruism as independent variables.

In the first step *fear of negative evaluation* accounted for 52% of the variance and the model was significant [*F* (1,350) = 387.65, *p* < 0.001], with *fear of negative evaluation* as significant predictor (*β* = 0.72, *p* < 0.001).

By adding the *personality facets* as independent variable in the second step of the regression model, and by controlling the influence of the *fear of negative evaluation*, the predictive value of the second model increases to 61% (ΔR^2^ = 0.09; *F* (3,347) = 27.80, *p* < 0.001), with *fear of negative evaluation* (*β* = 0.56, *p* < 0.001), *social boldness* (*β* = −0.13, *p* = 0.001), *liveliness* (*β* = −0.16, *p* < 0.001) and *organization* (*β* = −0.15, *p* < 0.001) as significant predictors.

In the third step, *altruism* was added to the regression model, resulting in an increase of the predictive value of the model to 63% (ΔR^2^ = 0.017; *F* (1,346) = 15.81, *p* < 0.001), after controlling the influence of *the fear of negative evaluation* and *personality facets*, with *altruism* (*β* = −0.14, *p* < 0.001), *fear of negative evaluation* (*β* = 0.55, *p* < 0.001), *social boldness* (*β* = −0.14, *p* < 0.001), *liveliness* (*β* = −0.12, *p* = 0.002) and *organization* (*β* = −0.12, *p* = 0.003) as significant predictors. As it can be observed from the results of the regression analysis, the power of liveliness and organization to predict the level of social interaction anxiety decreases after altruism is introduced as an independent variable in the model.

Therefore, the above-mentioned psychological constructs have been introduced into the SEM model. Taking into account the values provided by the hierarchical regression analysis, the following hypotheses regarding the relationships between the measured constructs were formulated:

*H7*: Fear of negative evaluation is positively associated with social interaction anxiety.

*H8*: Fear of negative evaluation is negatively associated with social boldness, liveliness, organization, and altruism.

*H9*: Social boldness is positively associated with liveliness.

*H10*: Liveliness is positively associated with organization.

*H11*: Organization is positively associated with altruism.

*H12*: Social boldness, liveliness, organization, and altruism are negatively associated with social interaction anxiety.

*H13*: Social boldness, liveliness, organization, and altruism play a serial mediating role in the association between fear of negative evaluation and social interaction anxiety.

Table 6 shows the HEXACO items measuring social boldness, liveliness, and organization together with alpha reliability (α), composite reliability (CR) and average variance extracted (AVE) for each subscale. All of the item loadings are above the accepted threshold of.60, and validity coefficients also exceeded the generally accepted threshold.

**Table 6:** Items loadings, composite reliability (CR), average variance extracted (AVE), and alpha Cronbach (α) for the scales measuring social boldness, liveliness, and organization.

Personality facets	Item loading	CR	AVE	*α*
Social boldness (Sb)		0.82	0.54	0.71
1. I rarely express my opinions in group meetings. (R)	0.737			
2. In social situations, I’m usually the one who makes the first move.	0.764			
3. When I’m in a group of people, I’m often the one who speaks on behalf of the group.	0.694			
4. I tend to feel quite self-conscious when speaking in front of a group of people.(R)	0.757			
Liveliness (Liv)		0.82	0.57	0.74
1. I am energetic nearly all the time.	0.713			
2. On most days, I feel cheerful and optimistic.	0.834			
3. People often tell me that I should try to cheer up.(R)	0.765			
4. Most people are more upbeat and dynamic than I generally am.(R)	0.706			
Organization (Org)		0.82	0.53	0.70
1. I clean my office or home quite frequently.	0.649			
2. I plan ahead and organize things, to avoid scrambling at the last minute.	0.709			
3. People often joke with me about the messiness of my room or desk.(R)	0.779			
4. I sometimes feel that I am a worthless person. (R)	0.784			

Items indicated with R are reverse-keyed items.

Table 7 presents the correlation matrix between social interaction anxiety, fear of negative evaluation, social boldness, liveliness, organization, and altruism.

**Table 7:** Correlation matrix between social interaction anxiety, fear of negative evaluation, social boldness, liveliness, organization, and altruism.

	Variables	*M*	SD	1	2	3	4	5
1	Social interaction anxiety (SIA)	0.87	0.82					
2	Fear of negative evaluation (FNE)	0.60	0.67	0.725^**^				
3	Social boldness (Sb)	3.61	0.68	−0.422^**^	−0.305^**^			
4	Liveliness (Liv)	3.92	0.67	−0.515^**^	−0.412^**^	0.479^**^		
5	Organization (Org)	4.22	0.62	−0.423^**^	−0.314^**^	0.242^**^	0.360^**^	
6	Altruism (Alt)	4.16	0.68	−0.370^**^	−0.239^**^	0.106^*^	0.322^**^	0.325^**^

*N* = 352; Social boldness, Liveliness, Organization = HEXACO Personality facets; Altruism = HEXACO Interstitial personality facet. *p < 0.05. **p < 0.01.

In the hypothesized model, fear of negative evaluation was entered as the independent variable (IV), social interaction anxiety was entered as the dependent variable (DV), social boldness was entered as first mediator (M1), liveliness was entered as second mediator (M2), organization was entered as third mediator (M3), and altruism was entered as fourth mediator (M4). According to this model, there were six possible pathways linking fear of negative evaluation to social interaction anxiety – one direct path and five indirect paths. As expected, the direct path connected fear of negative evaluation to social interaction anxiety. The first indirect pathway was through social boldness (M1), the second indirect pathway was through liveliness (M2), the third indirect pathway was through organization (M3) and the fourth indirect pathway was through altruism (M4). The fifth indirect pathway was through social boldness (M1), liveliness (M2), organization (M3) and altruism (M4), in serial. Not all the values of the hypothesized model reached the suggested values, indicating that the model is not adequate (Table 8).

**Table 8:** Goodness-of-fit of the invalidated model.

Fit index	X^2^/df	IFI	CFI	TLI	GFI	NFI	RFI	AGFI	SRMR	RMSEA
Suggested value	0–3	> 0.90	> 0.95	> 0.95	> 0.95	> 0.95	> 0.90	> 0.90	< 0.08	< 0.06
Values of this study	5.90	0.97	0.97	0.88	0.98	0.97	0.86	0.88	0.036	0.118

### 6.4. Hypotheses tested

As can be seen from Table 9, the hypotheses H7 to H12 and the relationships paths were supported by the data. Therefore:

- Fear of negative evaluation significantly and positively predicted social interaction anxiety (*β* = 0.56, *p* < 0.001), hence H7 was verified;
- Fear of negative evaluation significantly and negatively predicted social boldness (*β* = −0.30, *p* < 0.001), liveliness (*β* = −0.29, *p* < 0.001), and organization (*β* = −0.20, *p* < 0.001) but *not* altruism (*β* = −0.07, *p* = 0.127), therefore H8 was partially supported;
- Social boldness was positively associated with liveliness (*β* = 0.39, *p* < 0.001), liveliness was positively associated with organization (*β* = 0.28, *p* < 0.001) and organization was positively associated with altruism (*β* = 0.24, *p* < 0.001) therefore H9, H10 and H11were supported;
- Social boldness (*β* = −0.15, *p* < 0.001), liveliness (*β* = −0.13, *p* = 0.002), organization (*β* = −0.12, *p* = 0.003) and altruism (*β* = −0.14, *p* < 0.001) were negatively associated with social interaction anxiety, hence H12 was supported.

**Table 9:** The test results of path relationships between fear of negative evaluation, social interaction anxiety, social boldness, liveliness, organization, and altruism.

Hypothesis	Path	Unstandard estimates	S.E.	C.R.	Sig.	Standard estimates	LL 95%CI	UL95%CI	Hypothesis test
H7	FNE--->SIA	0.453	0.030	15.250	***	0.559	0.478	0.628	Supported
H8	FNE--->Sb	−0.255	0.042	−5.991	***	−0.305	−0.403	−0.203	Partially supported
	FNE--->Liv	−0.241	0.038	−6.301	***	−0.294	−0.387	−0.200	
	FNE--->Org	−0.152	0.041	−3.729	***	−0.200	−0.312	−0.084	
H9	Sb--->Liv	0.382	0.046	8.354	***	0.390	0.297	0.468	Supported
H10	Liv--->Org	0.258	0.050	5.180	***	0.278	0.165	0.397	Supported
H11	Org--->Alt	0.264	0.058	4.556	***	0.240	0.118	0.358	Supported
H12	Sb--->SIA	−0.144	0.036	−3.987	***	−0.149	−0.246	−0.065	Supported
	Liv--->SIA	−0.125	0.041	−3.084	0.002	−0.127	−0.211	−0.046	
	Org--->SIA	−0.130	0.039	−3.348	***	−0.122	−0.191	−0.051	
	Alt--->SIA	−0.138	0.034	−4.036	***	−0.142	−0.224	−0.062	
-	Liv--->Alt	0.240	0.054	4.470	***	0.235	0.124	0.347	-

FNE, Fear of negative evaluation; SIA, Social interaction anxiety: Sb., Social boldness; Liv., Liveliness; Org., Organization; Alt., Altruism.

All these results allow us to conclude that fear of negative evaluation has an indirect effect on social interaction anxiety through the mediation of social boldness, liveliness, and organization, separately. In this model, fear of negative evaluation does not have an indirect effect on social interaction anxiety through altruism.

**Figure 2:**
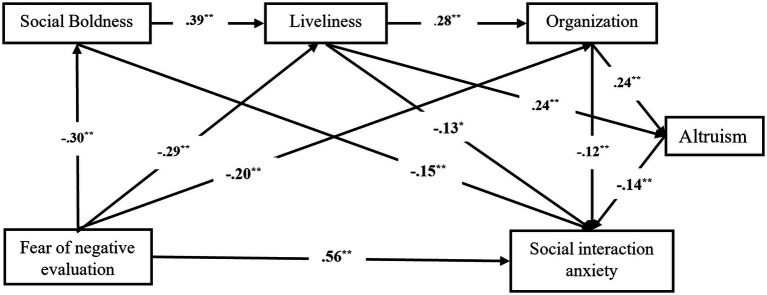
The path diagram of the relationships between fear of negative evaluation, social boldness, liveliness, organization, altruism, and social interaction anxiety.

The decrease in the power of liveliness and organization to predict the level of social interaction anxiety after altruism was introduced as an independent variable in the regression analysis suggested modifying the model by introducing altruism as a moderator variable between liveliness, organization, and social interaction anxiety. The relationships between variables were modified as illustrated in Figure 2 and the new model was tested. All the values of the new model reached the suggested values, which is an indicator that that the model is appropriate (Table 10).

**Table 10:** Goodness-of-fit of the second structural model.

Fit index	X^2^/df	IFI	CFI	TLI	GFI	NFI	RFI	AGFI	SRMR	RMSEA
Suggested value	0–3	> 0.90	> 0.95	> 0.95	> 0.95	> 0.95	> 0.90	> 0.90	< 0.08	< 0.06
Values of this study	2.18	0.99	0.99	0.97	0.99	0.99	0.95	0.95	0.022	0.058

These results invalidated the serial mediation hypothesis (H13) which stated that social boldness, liveliness, organization, and altruism play a serial mediating role in the association between fear of negative evaluation and social interaction anxiety. The bootstrap analysis of the significance test of the intermediate model effects in the second structural model is presented in Table 11.

**Table 11:** Bootstrap analysis of the significance test of the intermediate model effects in the second model.

	Path	Standard indirect effect	Unstandardized indirect effect	*p*	Bootstrap (95% CI)	
					Lower Limit	Upper Limit
Intermediary effect	FNE--->Sb--->SIA	0.045	0.037	<0.001	0.016	0.068
	FNE--->Liv--->SIA	0.037	0.030	0.002	0.010	0.059
	FNE--->Org--->SIA	0.024	0.020	0.001	0.006	0.042
	FNE--->Sb--->Liv--->Org--->SIA	0.003	0.003	0.001	0.001	0.007
Total intermediation effect		0.109	0.090		0.033	0.176
	Liv--->Alt--->SIA	−0.033	−0.033	< 0.001	−0.068	−0.013
	Liv--->Org--->SIA	−0.033	−0.034	0.001	−0.067	−0.013
	Liv--->Org--->Alt	0.067	0.068	< 0.001	0.032	0.128
	Org--->Alt--->SIA	−0.036	−0.036	0.001	−0.076	−0.014
	Liv--->Org--->Alt--->SIA	−0.009	−0.009	0.001	−0.022	−0.003
Total intermediation effect		−0.044	−0.044		−0.201	−0.085

FNE, Fear of negative evaluation; SIA, Social interaction anxiety: Sb., Social boldness; Liv., Liveliness; Org., Organization; Alt., Altruism.

The pathway FNE--->Sb--->Li--->Org--->SIA was also tested, and the following results were obtained: *β* = 0.003, BootLL = 0.001, BootUL = 0.007, *p* < 0.001. Based on these results, it can be stated that social boldness, liveliness, and organization play a serial mediating role in the association between fear of negative evaluation and social interaction anxiety.

The new pathway Liv--->Org--->Alt--->SIA has been tested and found to be significant: *β* = −0.009, BootLL = −0.022, BootUL = −0.003, *p* = 0.001.

After correlating all these results, it can be concluded that the model illustrated in Figure 2 is a moderated mediation model.

## 7. Discussion

This study aimed to examine the potential mediating effects of HEXACO personality traits on the relationship between fear of negative evaluation and social interaction anxiety. The preliminary analysis indicates that fear of negative evaluation has a positive association with social interaction anxiety and a negative association with extraversion, conscientiousness, and altruism. The relationship between fear of negative evaluation and social interaction anxiety was found to be fully mediated by the serial mediation pathway *via* extraversion, conscientiousness, and altruism. Also, the individual pathways between fear of negative evaluation and each of the mediators and social interaction anxiety were significant.

After validating the previous model, the potential mediating effects of the facets that make up extraversion (social self-esteem, social boldness, sociability and liveliness) and conscientiousness (organization, diligence, perfectionism and prudence) were tested. Each of the individual pathways between fear of negative evaluation and social boldness, liveliness and organization and social interaction anxiety turned out to be significant. The analysis has shown that the relationship between fear of negative evaluation and social interaction anxiety was fully mediated by the serial mediation pathway *via* social boldness, liveliness, and organization. The individual pathway between fear of negative evaluation, altruism and social interaction anxiety was not significant. The testing of the path relationships has shown the presence of significant direct relationships between (1) liveliness, organization, and altruism and (2) between altruism and social interaction anxiety; therefore, the moderating effect of altruism in the relationship between liveliness, organization, and social interaction anxiety was highlighted. Taking all these results into consideration, it can be stated that the relationship between fear of negative evaluation and social interaction anxiety is a moderated serial mediation pathway *via* social boldness, liveliness, organization, and altruism.

The levels of fear of negative evaluation and social interaction anxiety of the students included in the sample are extremely low. As military students, training and interacting at platoon level is likely to develop their ability to initiate and develop effective and rewarding interpersonal relationships. However, certain aspects of community life can generate fear of negative evaluation, especially in first-year students, such as those included in this study’s sample: insecurity about how they are perceived by others, the desire to be liked, appreciated and approved of, the desire to project a positive image, etc. They may experience episodes of social anxiety when having to speak in front of a group or a superior officer, isolate themselves in groups of friends to feel safe in platoons or battalions, feel anxious at social events or have difficulty initiating dyadic relationships, etc. The cadets sampled in this study are in the midst of a peer socialization process that is, by its nature, a comparative process in which cadets constantly adjust their behavior to match not only organizational norms but also those of their peers (Rubin and Burgess, 2001). They live on campus under the constant gaze of dozens of people ready to detect any deviation from the norm and correct it; in this context, peer relationships act as a social control factor that can generate negative evaluations of one’s own behavior, determined by how one is judged/appreciated by others (Perrot et al., 2012).

The strong mediating effect of extraversion on the relationship between fear of negative evaluation and social interaction anxiety was predictable because the influence of extraversion on various psychological constructs is well documented in many studies. Naturally, people with a high level of extraversion have a positive self-image, have a high level of self-confidence that helps them interact easily with people and groups, and feel comfortable in social gatherings; the higher their level of extraversion, the more they want to be the center of attention (Ashton and Lee, 2007). Two facets of extraversion have a mediating effect between fear of negative evaluation and social interaction anxiety: social boldness and liveliness. Individuals with a high level of social boldness are not only comfortable, but also very confident in social situations, are eager to assume leadership positions, speak easily in groups and approach strangers with confidence. People with a high level of liveliness are very energetic, optimistic, and enthusiastic. Therefore, even if an extroverted person experiences a certain degree of fear of being negatively evaluated by others, they will overcome it, thus lowering their level of anxiety in social interactions.

The second mediator in the first model analyzed in this paper is conscientiousness; it is a mediator with a weaker effect than extraversion, but nevertheless significant. Individuals with a high level of conscientiousness have a strong tendency to organize their time, activities, and environment, to achieve their goals through hard work, discipline, and patience. These individuals aspire to perfection in solving their tasks, often take on challenging goals, and carefully weigh alternatives and consequences when making decisions (Ashton and Lee, 2007). Because they have a natural tendency to adhere to socially prescribed norms, especially those pertaining to impulse control, these individuals can avoid anxiety in ambiguous social situations and orient themselves in these situations according to social norms that they are very familiar with (Bogg and Roberts, 2013). In general, a conscientious person will maintain a high level of cognitive control, including over the process of forming cognitive biases (Lonigan and Vasey, 2009) and the rumination that underlies anxiety (Noda et al., 2022; Liu et al., 2023a). As mentioned earlier, of all four facets of conscientiousness, only organization had a mediating effect between fear of negative evaluation and social interaction anxiety. A person with a strong tendency toward organization constantly seeks to maintain order, both in the way they approach tasks – in a highly structured way – and physically, where they rigorously maintain cleanliness and order.

The third mediator is altruism, described by the HEXACO authors as a person’s tendency to have sympathy and compassion for other people, to avoid hurting others, and to be generous toward them (Ashton and Lee, 2007). Altruism can determine a person to offer help and support to people in difficult situations, despite fear of negative evaluation or various negative feelings generated by social anxiety. In this way, a person can create a social network that acts over time as a factor in reducing social anxiety, simultaneously reducing the loneliness and isolation associated with the latter. The desire to help can lead to overcoming difficulties in interpersonal communication and the stress associated with fear of negative evaluation. In the case of our sample, the intense training program, consisting of military and academic training modules, creates situations where stress can reach peak levels, triggering intense emotional reactions; in these cases, the cadets’ behavior can become problematic, especially under the influence of feelings of deep insecurity. In this case, cadets are taught, through various methods, to provide emotional and instrumental support and to show empathy, sympathy, and compassion toward their colleagues. These are manifestations of altruism and each cadet learns and exercises them according to their own capabilities and dispositions. However, for the cadets sampled in this research, altruism is a significant mediator in the relationship between fear of negative evaluation and social interaction anxiety.

## 8. Practical implications

The analysis of the links between personality traits, fear of negative evaluation and social interaction anxiety facilitates the understanding of individualized risk trajectories and allows the creation of personalized interventions. Personality traits are involved in a multitude of cognitive and socio-emotional skills that can be learned, trained, and developed through education, so that fear of negative evaluation and social interaction anxiety do not manifest in the individuals’ behaviors. For example, Glinski and Page (2010) observed significant changes in the behavior of individuals with clinical levels of social anxiety. These people were part of a group undergoing cognitive-behavior therapy that addressed interpersonal issues. The authors argue that these changes were not due to alterations in the personality traits, but to modifications in their behavioral expression; however, any intervention that modifies the behaviors associated with the personality traits in order to increase the individual’s coping capacity is desirable.

In terms of extraversion, interventions can focus on the development of skills that belong to its facets: social self-esteem, social boldness, and sociability. Young people can learn to improve their self-esteem (Liu X. et al., 2022), to cultivate positive self-regard and self-beliefs (Hulme et al., 2012; Golde et al., 2023), self-compassion (Werner et al., 2012; Liu et al., 2020; Holas et al., 2023), self-efficacy, optimism, hope and resilience (Fatima et al., 2017; Serrano et al., 2021; Hui et al., 2022), emotional intelligence (Kahraman, 2022) and coping strategies (Takács et al., 2021).

Liu et al. (2023b) show that, beyond traditional approaches in the treatment of anxiety disorders, digital mental health interventions prove to be very effective due to low costs, rapid diagnosis, effective treatment and positive effects. Current digital interventions – web-based or computer-based programs, mobile applications, artificial intelligence based chatbots, virtual reality tools – can be perfected according to the demands and needs of students but also of stakeholders (country, society, colleges, and families).

In their studies, Cao and Liu (2023) and Cao (2023) concluded that practicing sports and sleeping have a positive effect on cognitive achievement, but time spent on homework, surfing the Internet, watching TV has a negative effect on it, when using depression symptoms as mediators. Thus, students can learn ways to optimally manage their time in the various tasks and activities specific to campus life in order to avoid anxiety or depression symptoms build-up.

Finnerty et al. (2021) believe that students should be offered certain extracurricular activities in which they can engage according to their preferences, depending on their personality traits, in order to increase their well-being. According to their research findings, students higher in conscientiousness or extraversion or emotional stability would prefer physical activities, while students higher in openness to experience would prefer to engage in journaling, playing a musical instrument, or singing.

As for the trait of conscientiousness, it encompasses socio-emotional skills that can also be learned: self-confidence, self-regulation, self-discipline, and sensitivity to fairness (Nasti et al., 2023).

Altruism can be developed in specific ways: through activities that involve volunteering and cooperation, by rewarding altruistic actions, by modeling caring behaviors (altruistic role models), through activities that develop individuals morally, by developing empathy, and by understanding social norms of reciprocity and social responsibility (Batson, 2011).

## 9. Limitations and future directions

Although the data of this study were processed by rigorous structural modeling techniques, the cross-sectional data does not allow the specification of causal relationships; for this, longitudinal or experimental studies are needed to allow a much clearer identification of causality and mutual influences between variables. All responses were collected by self-report procedures which are generally affected by social desirability bias; furthermore, all problems associated with “common method variance” are valid. However, since the tested models demonstrated a very good fit, and the correlations among the construct were not high, the issue of common method variance is not very problematic.

Another limitation of this study is given by the fact that it uses observed variables (a simple average of scale items). This approach is correct when the observed variables are entered into a regression analysis to test for mediation, which was the first stage in the statistical processing of our data. But statisticians usually recommend mediation testing by using latent variables in structural equation modeling. Latent variable models are capable of fitting the data better because they are using more parameter estimates than observed variable models; moreover, the latter tend to underestimate the amount of variation that is explained by the mediating variable. Nevertheless, while the latent variable models are able to produce more accurate estimates, the observed variable models are able to produce more precise estimates. Another element that must be considered is the reliability level of the measures used in the research: “As reliability decreases, both approaches become more troublesome. Even with an alpha of 0.7, which is typically considered an acceptable level of reliability in our field, observed variable approaches greatly underestimate path coefficients and can produce highly inflated Type I error rates, and latent variable approaches lose considerable power and (especially with large effect sizes) can occasionally yield wildly inaccurate estimates.[…] not only are observed variable analyses potentially biased, but latent variable analyses are potentially unstable” (Ledgerwood and Shrout, 2011, p. 15). Taking into account all this information, we consider that the first tested model has a high degree of validity (few variables, high reliability of the measures), while the second model requires a latent variables analysis for a definitive conclusion regarding its accuracy.

Although the research sample has not been established by a specific sampling procedure, but is rather a convenience sample, it can still be considered representative for the military student population, which is characterized by a high level of homogeneity. Numerous selection criteria are successively applied to candidates during admission to the military academies, in a cascade: minimum and maximum age limit, physical and clinical standards, sports scales, psychological and personality tests, assessment of specialized knowledge and evaluation of language skills. Subsequently, the military organizational culture, and the military lifestyle imbues future officers with a common set of values. All these result in a high level of homogeneity of the military student population, as compared to other populations.

As future research directions, we propose an examination of the relationships between fear of negative evaluation and social interaction anxiety and the other personality traits and facets measured by the HEXACO inventory. In the course of processing the data collected from the students, other significant relationships emerged that suggest the existence of a more complex model in which it is possible to introduce more traits or facets and to test complex mediating and moderating relationships between all the measured constructs.

## 10. Conclusion

The results presented in this paper pave the way for understanding the role of personality traits in mediating the relationship between fear of negative evaluation and social interaction anxiety in a social environment characterized by discipline, uniformity, and rigor, where any behavioral deviation is noticed. In the context of the present work, the analysis of personality traits of individuals in relation to social anxiety and specifically to the fear of negative evaluation can contribute to the identification of ways to stimulate desirable behaviors in the military organization, which is typified by compliance, conformity and predictability.

Military education and training are largely guided by traditional values, rules, and practices, repeatedly imposed to individual and group levels. In order to update and improve the educational approaches on scientific basis, a more comprehensive understanding of the cadets’ behaviors and attitudes in specific social contexts is needed. Since the professional activity of an officer involves constant social interactions and his/her permanent exposure in front of subordinates, it is important for the cadets to develop their ability to appropriately manage social interactions at any level of the organization.

## Data availability statement

The raw data supporting the conclusions of this article will be made available by the authors, without undue reservation.

## Ethics statement

Ethical review and approval was not required for the study on human participants in accordance with the local legislation and institutional requirements. The studies were conducted in accordance with the local legislation and institutional requirements. The participants provided their written informed consent to participate in this study.

## Author contributions

CM: Conceptualization, Formal analysis, Investigation, Methodology, Project administration, Supervision, Writing – original draft, Writing – review & editing. ȘB: Investigation, Writing – original draft, Writing – review & editing. FM-B: Investigation, Writing – original draft, Writing – review & editing.
